# Reduced Mortality in Maintenance Haemodialysis Patients on High versus Low Dialysate Magnesium: A Pilot Study

**DOI:** 10.3390/nu9090926

**Published:** 2017-08-23

**Authors:** Christoph Schmaderer, Matthias C. Braunisch, Yana Suttmann, Georg Lorenz, Dang Pham, Bernhard Haller, Susanne Angermann, Julia Matschkal, Lutz Renders, Marcus Baumann, Jürgen R. Braun, Uwe Heemann, Claudius Küchle

**Affiliations:** 1 Department of Nephrology, Klinikum rechts der Isar, Technische Universität München, Ismaninger Straße 22, 81675 Munich, Germany; Christoph.Schmaderer@mri.tum.de (C.S.); Matthias.Braunisch@mri.tum.de (M.C.B.); Yana.Suttmann@mri.tum.de (Y.S.); Georg.Lorenz@mri.tum.de (G.L.); D.dang.pham@googlemail.com (D.P.); Susanne.Angermann@mri.tum.de (S.A.); Julia.Matschkal@mri.tum.de (J.M.); Lutz.Renders@mri.tum.de (L.R.); Marcus.Baumann@klinikum-ansbach.de (M.B.); Uwe.Heemann@mri.tum.de (U.H.); 2 Institute of Medical Statistics and Epidemiology (IMSE), Klinikum rechts der Isar, Technische Universität München, Ismaninger Straße 22, 81675 Munich, Germany; Bernhard.Haller@tum.de; 3 Section of Nephrology, Klinikum Ansbach, Escherichstraße 1, 91522 Ansbach, Germany; 4 Praxen Dr. Braun, Dialysis Center Dingolfing, Aitrachstraße 5, 84130 Dingolfing, Germany; jrbraun@gmx.de

**Keywords:** chronic haemodialysis, dialysate magnesium, cardiovascular mortality, ionized magnesium, matched patients

## Abstract

Background: Although low magnesium levels have been associated with an increased mortality in dialysis patients, they are kept low by routinely-used dialysates containing 0.50 mmol/L magnesium. Thus, we investigated the impact of a higher dialysate magnesium concentration on mortality. Methods: 25 patients on high dialysate magnesium (HDM) of 0.75 mmol/L were 1:2 matched to 50 patients on low dialysate magnesium (LDM) of 0.50 mmol/L and followed up for 3 years with regards to all-cause and cardiovascular mortality. Patients were matched according to age, gender, a modified version of the Charlson Comorbidity Index (CCI), and smoking status. Results: During the follow-up period, five patients died in the HDM and 18 patients in the LDM group. Patients in the HDM group had significantly higher ionized serum magnesium levels than matched controls (0.64 ± 0.12 mmol/L vs. 0.57 ± 0.10 mmol/L, *p* = 0.034). Log rank test showed no difference between treatment groups for all-cause mortality. After adjustment for age and CCI, Cox proportional hazards regression showed that HDM independently predicted a 65% risk reduction for all-cause mortality (hazard ratio 0.35, 95% confidence interval [CI]: 0.13, 0.97). Estimated 3-year probability of death from a cardiovascular event was 14.5% (95% CI: 7.9, 25.8) in the LDM group vs. 0% in the HDM group. Log rank test found a significant group difference for cardiovascular mortality (χ^2^ = 4.15, *p* = 0.042). Conclusions: Our data suggests that there might be a beneficial effect of an increased dialysate magnesium on cardiovascular mortality in chronic dialysis patients.

## 1. Introduction

In patients with chronic kidney disease, cardiovascular disease is the major cause of morbidity and mortality [1,2,3]. A contributing factor may be low serum magnesium concentrations [4,5,6,7], while high concentrations of serum magnesium were associated with a lower rate of mortality.

De Roij et al. observed an increase in baseline serum magnesium of 0.10 mmol/L with a hazard ratio of 0.86 for all-cause mortality, 0.73 for cardiovascular mortality, and 0.76 for sudden cardiac death after a mean follow-up of 3.1 years [8]. In univariate analysis, this has also been confirmed for 1-year mortality risk [9] as well as in cohorts with patients on peritoneal dialysis or with chronic kidney disease [10,11]. Nonetheless, deficiencies of ionized serum magnesium in patients on haemodialysis have been described ever since 1993 [12].

The serum magnesium concentration is linked to dietary magnesium intake [13]. However, adherence to dietary recommendations in haemodialysis patients is low and magnesium rich-foods are also rich in potassium [14,15]. When combined with the loss of magnesium due to food processing, hypomagnesaemia cannot be sufficiently regained by nutrition [16]. Thus, in patients on maintenance haemodialysis, serum magnesium levels are primarily determined by the dialysate magnesium concentration.

At present, a dialysate magnesium concentration of 0.50 mmol/L is routinely prescribed. This low dialysate magnesium (LDM) concentration inevitably induces post dialytic hypomagnesaemia [17]. On the other hand, increasing dialysate magnesium is known to increase serum magnesium [9,17]. We have recently demonstrated that a long term substitution of magnesium by a higher dialysate magnesium concentration of 0.75 mmol/L increases serum magnesium to stable levels within the higher normal range (1.02 mmol/L total magnesium) without any hints for health concerns [17]. This is important, bearing in mind that excessive serum magnesium levels above 1.27 mmol/L have been associated with an increased odds ratio for cardiovascular death in a single cohort with dialysis patients, which was, however, attributed to low PTH levels by the authors [5].

The ability of high magnesium levels to compensate for high phosphate levels and, via this pathway, reduce cardiovascular risk [18], necessitates further investigation into magnesium substitution with regard to the prognosis of patients on haemodialysis. Only recently, such an intervention study has been proposed [15].

We present for the first time the outcome of a longitudinal matched pair study in patients on maintenance haemodialysis treated with low (0.50 mmol/L) or high (0.75 mmol/L) dialysate magnesium concentration.

## 2. Materials and Methods

### 2.1. Study Design

The study was conducted in an observational case-control design. Patients of the high dialysate magnesium (HDM) group were recruited from a dialysis center near Munich, where a standard dialysate magnesium concentration of 0.75 mmol/L had been chosen for all patients based on the assumptions outlined before. Matches were recruited in dialysis centers with a dialysate magnesium concentration of 0.50 mmol/L in Munich and the greater Munich area. The small sample size of 25 patients in the HDM group was owed to the low number of available patients in this subgroup; to increase the overall sample size and minimize the standard deviation at least in the LDM group, a 1:2 matching was performed. The study was conducted in accordance with the Declaration of Helsinki, and the protocol was approved by the Medical Ethics Committee of the Klinikum rechts der Isar, Technical University Munich (no. 1667/06, date: 5 December 2006). All participants gave their written informed consent for inclusion before they participated in this study.

### 2.2. Study Conduct

Baseline clinical data including medical history and chemistry were obtained at study entry. To perform the measurement of ionized magnesium, blood samples were taken before a midweek dialysis session before anticoagulation was begun to avoid interference with heparin or citrate [19]. In haemodialysis patients, total magnesium levels tend to underestimate magnesium deficits [19,20]. We therefore chose to measure ionized magnesium levels as it represents the biological active form of magnesium and gives a more reliable information on the patient’s magnesium status. Measurements of ionized magnesium and ionized calcium were performed with an ion-selective electrode (Magnesiometer Type CRT 8, Nova Biomedical, Waltham, MA, USA).

Dialysate magnesium concentration was increased to 0.75 mmol/L by using a commercially available dialysate mixture and supply system (CK Medizintechnik, Büren, Germany). The dialysate composition was regularly verified by a certified laboratory.

Blood for the analysis of ionized magnesium, ionized calcium, and high resolution C-reactive protein (hsCRP) was drawn at study entry. A threshold of plus/minus 3 months was accepted for the assessment of the other serum chemistry values in Table 1 that are taken quarterly, by routine, in German dialysis centers. HsCRP was assayed with latex-enhanced reagents (Siemens, Munich, Germany) on a BN ProSpec analyzer (Siemens) following the manufacturer’s instructions. Other serum chemistry values were performed in ISO-certified laboratories in the different dialysis centers and also drawn before a midweek dialysis session.

### 2.3. Inclusion and Exclusion Criteria

Patients were included if they were at least 18 years old, had been on dialysis for at least 90 days, and were willing to participate in the study. Patients were excluded if an ongoing infection, pregnancy, or malignant disease with a life expectancy of less than 24 months was present, or they were unwilling to participate.

### 2.4. Treatment Groups

**Figure A1:**
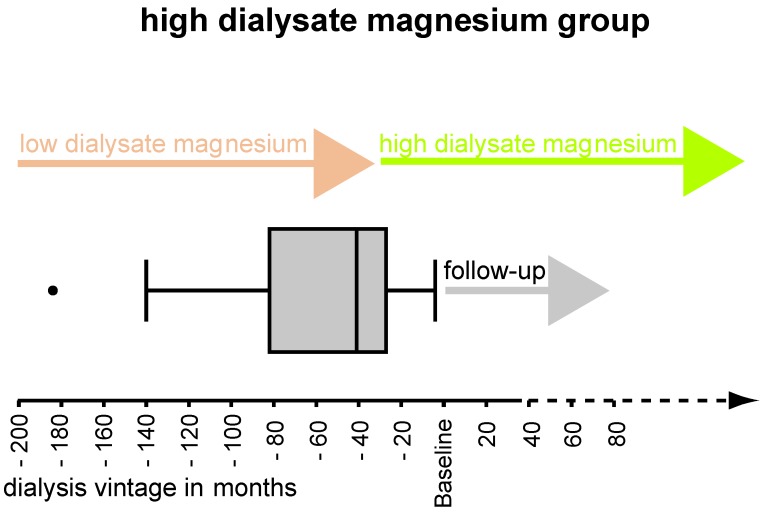
Box plot of the dialysis vintage in the high dialysate magnesium group. Median dialysis vintage of patients in the high dialysate magnesium group was 41.0 (30.0–96.5), minimum was 4 and maximum was 184 months. 17 (68.0%) of the patients were switched 31–33 months before baseline from low (0.50 mmol/L) to high dialysate magnesium (0.75 mmol/L). The dialysate magnesium concentration was not changed during the follow-up period.

Dialysate magnesium concentration was 0.75 mmol/L in the HDM group and 0.50 mmol/L in the LDM group. Patients were at least three months on dialysis with the respective magnesium concentration before baseline. The magnesium concentration was not changed during the follow-up period. Patients on HDM were on this bath for at least 4 months (see Figure A1).

### 2.5. Endpoint Analysis

All-cause and cardiovascular mortality were selected as primary outcome variables. Mortality was ascertained approximately 3 years (median: 1200 days) after baseline. Mortality events were documented form clinic reports, medical records, and interviews with the physicians in the dialysis centers. Cardiovascular mortality was coded if one of the following conditions was present: sudden cardiac death, defined as a sudden pulseless condition out of the hospital, or, if the event was unwitnessed, sudden cardiac death was suspected if the patient had been seen stable within the last 24 h preceding the event (*n* = 2); death due to a history of myocardial infarction defined as chest pain, dynamic troponin change, cardiogenic shock, or ECG with ST-elevation (*n* = 2); death due to heart failure (*n* = 4).

### 2.6. Matching

Patients were matched 1:2 using the case-control matching in SPSS. Patients were matched according to age, gender, Charlson Comorbidity Index (CCI), and smoking status. For age an inaccuracy of 2.5 years, and for CCI, an inaccuracy of the ordinal score of 1 was accepted. To evaluate and match patients’ comorbidity status, a modified version of the CCI designed for patients with end-stage renal disease was used [21]. The CCI assigns numerical weights to the comorbid conditions of atherosclerotic heart disease (1), congestive heart failure (3), cerebrovascular accident/transient ischaemic attack (2), peripheral vascular disease (2), dysrhythmia (2), other cardiac disease (2), chronic obstructive pulmonary disease (2), gastrointestinal bleeding (2), liver disease (2), cancer (2), and diabetes (1). It has been shown that the CCI categories are highly predictive and separating for survival in patients with end-stage renal disease, and therefore they are a valuable tool for the adjustment of comorbid conditions and outcome analysis [21].

### 2.7. Statistical Analysis

Categorical data are presented as frequencies and percentages. Continuous variables are expressed as mean ± standard deviation (SD) for normally distributed variables and as median and interquartile range (IQR, 25th–75th percentile) for variables with skewed distribution.

To test for differences between the matched treatment groups of HDM versus LDM, χ^2^ test was used for categorical variables and independent samples *t*-test or Mann-Whitney *U* test for continuous variables, as appropriate.

Survival curves for overall mortality were estimated by the Kaplan-Meier method. For the probability of cardiovascular events, cumulative incidence functions, considering deaths from other causes as competing risks, are presented. Hazards for overall mortality and a cause-specific hazard for cardiovascular mortality were compared between groups by the log-rank test. Univariate Cox proportional hazards regression analysis was performed for all-cause mortality. Adjusting for matching variables is recommended in a matched population to allow for a more precise effect estimation of the variable of interest [22]. Therefore, adjusted Cox proportional hazards regression was performed for all-cause mortality with the matching variables’ age and CCI that showed a significant univariate association with all-cause mortality. Additionally, BMI, LDL cholesterol, and Kt/V and total cholesterol that differed significantly and by trend, respectively, between the two groups, were examined with univariate Cox proportional hazards regression. Due to the presence of 23 events in the total group, we could only adjust for two variables. The two variables age and CCI with the lowest *p*-value in univariate analysis were selected for adjustment (Table A1). Due to the absence of cardiovascular mortality events, Cox proportional hazards regression could not be performed in this subgroup.

Median follow-up time was assessed using the reverse Kaplan-Meier method for estimation of potential follow-up time proposed by Schemper and Smith 1996 [23].

All statistical tests were two-sided and *p*-values < 0.05 were considered significant. Statistical analysis was performed using SPSS version 23.0 (SPSS Inc., Chicago, IL, USA) for Mac and R version 3.3.2. [24].

## 3. Results

25 patients on HDM were matched to 50 patients on LDM according to age, gender, CCI, and smoking status. Baseline characteristics did not differ significantly in terms of age, gender, dialysis parameters, blood pressure, laboratory values, and comorbidities except for BMI, ionized serum magnesium, LDL cholesterol, and by trend Kt/V and total cholesterol (Table 1, Table A1). The matching variables (i.e., age, gender, CCI, and smoking status), as well as the variables that differed significantly between the two groups (i.e., BMI, ionized serum magnesium, LDL cholesterol, and by trend Kt/V and total cholesterol), were examined by univariate Cox regression analysis for their predictive value of all-cause mortality. Only age, CCI, and Kt/V showed a univariate significant association with all-cause mortality (Table A1).

**Figure 1:**
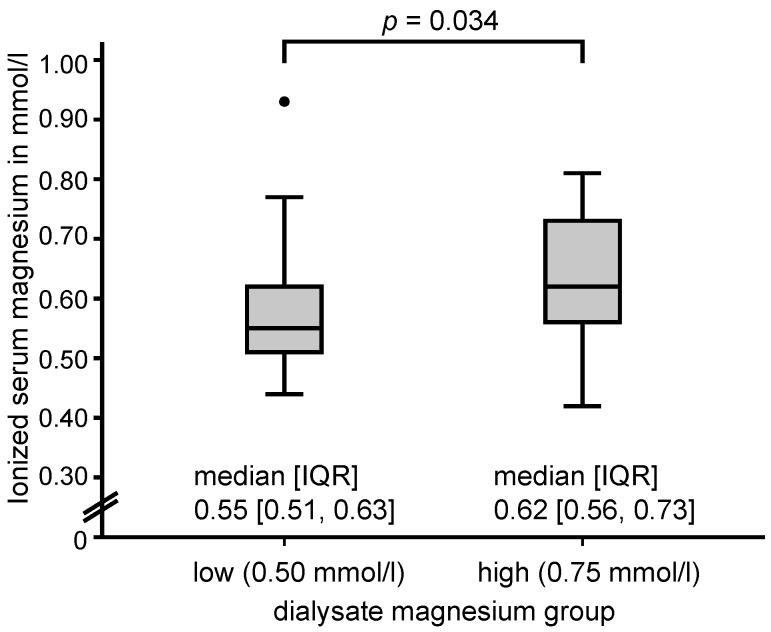
Box plot with median and interquartile range differences in ionized serum magnesium concentration in patients on low (0.50 mmol/L) and high (0.75 mmol/L) dialysate magnesium concentration, collected before a midweek dialysis session at study entry. IQR, interquartile range.

As expected, patients in the HDM group had a significantly higher ionized serum magnesium (0.64 ± 0.12 mmol/L, *n* = 25, corresponding to approx. 1.05 mmol/L total magnesium) compared to patients in the LDM group (0.57 ± 0.10 mmol/L, *p* = 0.034, *n* = 41) (Figure 1); mean difference was 0.06 (95% CI: 0.01, 0.12). In the HDM group, the minimum magnesium value was 0.42 mmol/L and the maximum magnesium value was 0.81 mmol/L. In the LDM group, the minimum and maximum magnesium values were 0.44 mmol/L and 0.93 mmol/L, respectively (see Figure 1). Four patients (16.0%) in the HDM and three patients (6.0%) in the LDM group, respectively, had ionized levels above 0.78 mmol/L. Furthermore, patients in the HDM group had a significantly higher BMI (28.1 (25.0–34.7) kg/m^2^ vs. 24.4 (22.2–29.2) kg/m^2^; *p* = 0.034), significantly lower LDL cholesterol (91.1 ± 39.4 mg/dL vs. 117.8 ± 36.6 mg/dL, mean difference −26.76 (95% CI: −47.36, −6.15); *p* = 0.012), and showed a trend for a higher Kt/V (1.53 ± 0.29 vs. 1.37 ± 0.43, mean difference 0.16 (95% CI: −0.01, 0.32); *p* = 0.068), and a lower total cholesterol (167.0 ± 48.2 mg/dL vs. 190.0 ± 48.5 mg/dL, mean difference −23.02 (95% CI: −48.56, 2.52); *p* = 0.08).

In the HDM group one (4%) and in the LDM group four (8%), patients were lost to follow-up. Reasons for loss to follow-up were: kidney transplantation (*n* = 3), relocation to another city (*n* = 1), and unknown cause (*n* = 1). In the HDM group, five patients (21%) died due to discontinuation of dialysis due to palliative reasons (*n* = 1), sepsis (*n* = 1), Merkel-cell carcinoma (*n* = 1), and unknown causes (*n* = 2). In the LDM group, 18 patients (39%) died due to sepsis (*n* = 5), heart failure (*n* = 4), sudden cardiac death (*n* = 2), history of myocardial infarction (*n* = 2), unknown causes (*n* = 2), chronic anemia due to hemorrhage and autoimmune hemolysis in the presence of osteomyelofibrosis (*n* = 1), pancreatic carcinoma (*n* = 1), and perioperatively (*n* = 1).

Univariate Cox proportional hazard regression was calculated with dialysate magnesium concentration as a covariate for all-cause mortality. In univariate analysis, magnesium concentration was not significantly associated with all-cause mortality (hazard ratio 0.54 (95% CI: 0.20, 1.46); *p* = 0.22) (Table 2). After adjusting for age and CCI, the dialysate magnesium was independently and significantly associated with all-cause mortality (hazard ratio 0.35 (95% CI: 0.13, 0.97); *p* = 0.044) (Table 2 and Table A2).

**Figure 2:**
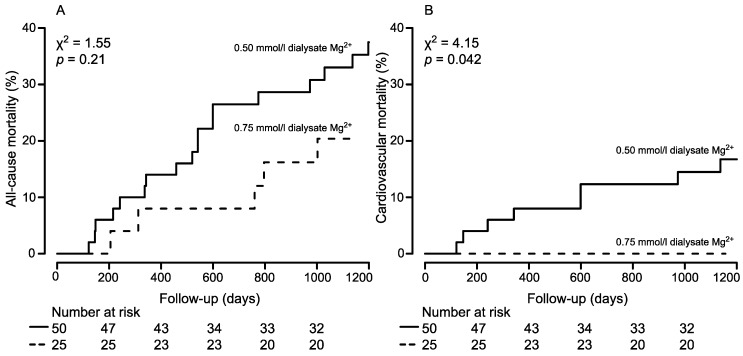
Cumulative all-cause mortality (**A**) and cardiovascular mortality (**B**) curves for patients stratified by low (0.50 mmol/L) and high (0.75 mmol/L) dialysate magnesium concentration, respectively. The number of patients of the individual groups involved in the analysis at 0, 200, 400, 600, 800, and 1000 days are shown below each graph.

The Kaplan-Meier estimates indicated that the all-cause mortality at approx. 3-years (1200 days) for 25 patients on HDM was 20.4% (95% CI: 2.7, 34.5) and for 50 patients on LDM was 33.0% (95% CI: 18.3, 45.1). The log rank test indicated that there was no statistically significant difference between the two groups for all-cause mortality rates (*p* = 0.21) (Figure 2A).

Because magnesium is particularly involved in the calcification processes, and therefore in cardiovascular morbidities, the cumulative incidence function for cardiovascular mortality was estimated: no cardiovascular death was observed in the 25 patients with HDM, and the estimated approx. 3-year (1200 days) probability for death from a cardiovascular event was 14.5% (95% CI: 7.9, 25.8) in the LDM group. The log rank test indicated a statistically significant difference between the cardiovascular mortality hazards of the two groups (*p* = 0.042) (Figure 2B).

## 4. Discussion

The association between higher serum magnesium levels and lower mortality in haemodialysis patients has been widely documented [7,8]. Higher magnesium levels even compensated the mortality risk of hyperphosphatemia in a large Japanese cohort [18]. In vitro evidence exists that magnesium prevents phosphate from complexing with calcium [25] and therefore prevents calcification of vascular smooth muscle cells. Magnesium deficits contribute to smooth muscle cell ossification, atherosclerosis [4,5,6], and endothelial dysfunction [10], and therefore to an increased cardiovascular mortality risk. It would thus be most interesting to investigate if higher dialysate magnesium concentrations would impact clinical parameters of arteriosclerosis, like, for example, the pulse wave velocity.

Albeit deficiencies of serum ionized magnesium in dialysis patients have been noted since 1993 [12], the commonly used dialysates contain only low-ionized magnesium concentrations of 0.50 mmol/L. This was based on the assumption that alimentary and drug derived magnesium would expose end-stage renal disease patients to excessive magnesium levels. Only recently, we revealed that LDM concentrations induce iatrogenic hypomagnesaemia, and that this condition is easily correctable by higher dialysate magnesium concentrates without causing any apparent harm [17].

Accordingly, we now again found that patients on a HDM of 0.75 mmol/L had significantly higher mean ionized serum magnesium levels than matched controls with a LDM of 0.50 mmol/L (Table 1, Figure 1). Now, and for the first time, an independent significant mortality risk reduction in the HDM group for all-cause mortality was observed, applying Cox proportional hazards regression adjusted for age and CCI. Additionally, we observed favorable cardiovascular mortality rates for patients on HDM (estimated approx. 3-year probabilities: 0% vs. 14.5%).

We could furthermore demonstrate that, after adjusting for well-known mortality risks like age and comorbidity status, the dialysate magnesium concentration was significantly associated with all-cause mortality. Taken together, these results suggest that higher dialysate magnesium leading to higher serum levels reduces the mortality risk compared to standard treatment with low dialysate magnesium.

Due to the possibly harmful effect of exaggerated magnesium levels reported by Sakaguchi et al. [5], we used a rough approximation to convert ionized magnesium into total magnesium (Total magnesium = ionized magnesium × 100)/61 [17]). When using this approximation, 1.27 mmol/L total magnesium is equivalent to 0.78 mmol/L of ionized magnesium. This threshold was exceeded in four patients of the HDM group. None of these four patients died. Nevertheless, this finding underlines the importance that a regular measurement of magnesium should be performed.

This case-control study is subject to all inherent limitations of such studies. In particular, unknown or unobserved variables could explain the group differences. However, to access the effect of dialysis magnesium concentration on the outcome, only a randomized controlled trial could guarantee an equal distribution of unknown disturbance variables, and it is highly desirable. Unfortunately, this approach has been hampered throughout the last years by missing safety data on HDM concentration and a complete lack of funding.

Furthermore, the sample size of the treatment group with HDM was relatively small. Patients of this group were recruited from a single dialysis center. Therefore, the results cannot be easily generalized and should be confirmed in larger studies. However, the data is consistent with hypotheses on magnesium in the context of mortality risk. Given the mortality event rate of 23, multivariable adjustment could only be performed for two variables. We also lacked cause-of-death details of four patients (two in each group). These patients were coded as death from non-cardiovascular cause. But it cannot be ruled out that these patients died due to cardiovascular events. Also, we lack data on all-cause and cardiovascular mortality in the dialysis unit before the increase of dialysate magnesium levels. While median follow-up was only 1200 days, given the overall 5-year survival rate in haemodialysis patients of 50% after the start of dialysis, reported by the European Dialysis and Transplant Association [26], we regarded a median follow-up of approx. 3 years acceptable at least for a primary investigation. The ongoing follow-up of our patients will hopefully further confirm the results of this work.

Despite the limitations of our small pilot study, we hope that this data will stimulate debate and might pave the path for larger randomized trials that are clearly needed in order to decrease mortality risk of our dialysis patients. In so doing, this will inevitably provide additional insights into the role of magnesium in cardiovascular disease.

In conclusion, this is the first study to demonstrate that HDM after adjustment for age and comorbidities independently predicts mortality and is associated with a more beneficial cardiovascular survival rate.

## Figures and Tables

**Table 1:** Baseline characteristics in patients with high (0.75 mmol/L) vs. low (0.50 mmol/L) dialysate magnesium.

	Dialysate Magnesium		
	High 0.75 mmol/L (*n* = 25)	Low 0.50 mmol/L (*n* = 50)	*p*-Value
Age (years)	66.3 (48.5–73.5)	67.9 (49.2–73.9)	0.86
Gender (males)	16 (64.0)	32 (64.0)	1.0
BMI (kg/m^2^)	28.1 (25.0–34.7)	24.4 (22.2–29.2)	0.034
Follow-up (days)	1121 (1047.5–1138.5)	1200 (550.8–1200.0)	0.06
Dialysis vintage (months)	41.0 (30.0–96.5)	47.0 (26.5–99.3)	0.86
Ultrafiltration rate per hour	563.6 ± 283.0	478.7 ± 263.5	0.37
Volume reduction per session (L)	1.9 ± 1.7	1.6 ± 1.2	0.37
Kt/V	1.53 ± 0.29	1.37 ± 0.43	0.07
Dialysis duration per session (h)	4.5 (4.0–4.5)	4.2 (4.0–4.5)	0.88
Systolic arterial pressure (mmHg)	137.7 ± 18.1	129.9 ± 27.0	0.20
Diastolic arterial pressure (mmHg)	66.7 ± 17.1	68.5 ± 13.3	0.63
Ionized magnesium in serum (mmol/L)	0.64 ± 0.12	0.57 ± 0.10	0.034
Phosphate (mmol/L)	1.53 (1.37–1.94)	1.75 (1.40–2.11)	0.16
Total calcium in serum (mmol/L)	2.28 ± 0.24	2.27 ± 0.20	0.89
Ionized calcium in serum (mmol/L)	1.22 ± 0.18	1.21 ± 0.13	0.86
Calcium x phosphate (mmol^2^/L^2^)	3.78 (2.87–4.51)	3.90 (3.14–4.74)	0.23
Alkaline phosphatase (U/L)	74 (56–90)	88 (66–113)	0.10
Intact parathyroid hormone (pg/mL)	152.48 (51.00–189.50)	191.10 (86.55–361.00)	0.11
High resolution CRP (mg/L)	0.68 (0.16–1.28)	0.55 (0.16–1.37)	0.94
Albumin (g/dL)	3.94 (3.78–4.33)	3.96 (3.70–4.10)	0.24
Blood urea nitrogen (mg/dL)	59.48 ± 16.61	63.23 ± 17.37	0.70
Hematocrit (%)	35.74 ± 4.29	34.69 ± 4.79	0.34
Haemoglobin (g/dL)	11.72 ± 1.35	11.61 ± 1.45	0.74
Total cholesterol (mg/dL)	167.0 ± 48.2	190.0 ± 48.5	0.08
HDL cholesterol (mg/dL)	41.0 (37.8–55.8)	45.0 (37.8–55.8)	0.21
LDL cholesterol (mg/dL)	91.1 ± 39.4	117.8 ± 36.6	0.012
Charlson Comorbidity Index (1–4)	1.0 (0–1.5)	1.0 (0–1.0)	0.27
Diabetes mellitus	16 (64.0)	24 (48.0)	0.23
History of myocardial infarction	7 (28.0)	12 (24.0)	0.78
Peripheral artery disease	9 (36.0)	14 (28.0)	0.60
Smoking (ever)	4 (16.0)	8 (16.0)	1.0

*n* (%) for categorical data, mean ± standard deviation or median [interquartile range] for continuous data, as appropriate. Comparison of groups by χ^2^ for categorical data, independent samples *t*-test or Mann-Whitney *U* Test for continuous data, as appropriate.

**Table 2:** Univariate and adjusted hazard ratios of high dialysate magnesium for all-cause mortality risk.

Predictor	Unadjusted		Adjusted *	
	Hazard Ratio (95% CI)	*p*-Value	Hazard Ratio (95% CI)	*p*-Value
High dialysate magnesium	0.54 (0.20, 1.46)	0.24	0.35 (0.13, 0.97)	0.044

* Adjusted for age and CCI. CI, confidence interval.

## Appendix A

**Table A1:** Univariate Cox proportional hazards regression of matching variables and variables differing between groups.

Predictor	Unit	Hazard Ratio (95% CI)	*p*-Value
Age *	1 year	1.06 (1.02, 1.10)	0.002
Gender	Male	1.92 (0.76, 4.88)	0.17
CCI *	1	2.75 (1.80, 4.20)	<0.001
Smoking status	Smoker	0.04 (0.00, 3.94)	0.17
BMI	1 kg/m^2^	1.04 (0.98, 1.11)	0.24
Kt/V	1	0.34 (0.12, 0.99)	0.047
Total cholesterol	1 mg/dL	0.99 (0.98, 1.00)	0.14
LDL cholesterol	1 mg/dL	0.99 (0.98, 1.01)	0.25

* selected for adjusted Cox proportional hazards regression model. CI, confidence interval.

**Table A2:** Variables in the equation for adjusted Cox proportional hazards regression.

Predictor	Unit	Regression Coefficient	Hazard Ratio (95% CI)	*p*-Value
Dialysate group	High dialysate magnesium	−1.06	0.35 (0.13, 0.97)	0.044
Age	1 year	0.04	1.04 (1.00, 1.09)	0.08
CCI *	1	0.96	2.60 (1.58, 4.28)	<0.001

* The hazard ratio is described for a difference of 1 out of the four score groups. CI, confidence interval.
